# IgM and IgG against *Plasmodium falciparum* lysate as surrogates of malaria exposure and protection during pregnancy

**DOI:** 10.1186/s12936-018-2331-4

**Published:** 2018-05-10

**Authors:** Alfredo Mayor, Carlota Dobaño, Augusto Nhabomba, Caterina Guinovart, Alfons Jiménez, Maria Nelia Manaca, Ruth Aguilar, Arnoldo Barbosa, Mauricio H. Rodríguez, Pau Cisteró, Lazaro M. Quimice, Clara Menéndez, John J. Aponte, Jaume Ordi, Chetan E. Chitnis, Pedro L. Alonso

**Affiliations:** 1 ISGlobal, Hospital Clínic-Universitat de Barcelona, Carrer Rosselló 153 (CEK Building), 08036 Barcelona, Spain; 2 Centro de Investigação em Saúde de Manhiça (CISM), Maputo, Mozambique; 3 CIBER Epidemiología y Salud Pública (CIBERESP), Madrid, Spain; 4 International Centre for Genetic Engineering and Biotechnology, New Delhi, India

**Keywords:** Malaria, Pregnancy, Heterogeneity, Antibody, Immunity

## Abstract

**Background:**

Difficulties to disentangle the protective versus exposure role of anti-malarial antibodies hamper the identification of clinically-relevant immune targets. Here, factors affecting maternal IgG and IgMs against *Plasmodium falciparum* antigens, as well as their relationship with parasite infection and clinical outcomes, were assessed in mothers and their children. Antibody responses among 207 Mozambican pregnant women at delivery against MSP1_19_, EBA175, AMA1, DBLα and parasite lysate (3D7, R29 and E8B parasite lines), as well as the surface of infected erythrocytes, were assessed by enzyme-linked immunosorbent assay and flow cytometry. The relationship between antibody levels, maternal infection and clinical outcomes was assessed by multivariate regression analysis.

**Results:**

Placental infection was associated with an increase in maternal levels of IgGs and IgMs against a broad range of parasite antigens. The multivariate analysis including IgGs and IgMs showed that the newborn weight increased with increasing IgG levels against a parasite lysate, whereas the opposite association was found with IgMs. IgGs are markers of protection against poor pregnancy outcomes and IgMs of parasite exposure.

**Conclusions:**

Adjusting the analysis for the simultaneous effect of IgMs and IgGs can contribute to account for heterogeneous exposure to *P. falciparum* when assessing immune responses effective against malaria in pregnancy.

**Electronic supplementary material:**

The online version of this article (10.1186/s12936-018-2331-4) contains supplementary material, which is available to authorized users.

## Background

*Plasmodium falciparum* infection during pregnancy, characterized by the accumulation of parasites in the intervillous spaces of the placenta, is a major disease that affects birth outcome in sub-Saharan Africa [1], causing up to 100,000 infant deaths annually [2]. In contrast to non-pregnant adults, pregnant women are at increased risk of malaria infection independently of previous acquisition of immunity [3]. In conditions of high malaria transmission, this susceptibility decreases with subsequent pregnancies [3], suggesting a parity-dependent acquisition of protective immunity. Understanding the targets, quality and quantity of the immune responses that pregnant women mount upon infection and their role, in protection against infection and its adverse effects in pregnancy (i.e., low newborn weight, stillbirth and maternal anaemia) is of critical importance to design effective vaccines specific for pregnant women [4].

Immunoglobulin G (IgG) antibodies against placental infected erythrocytes (IE) and VAR2CSA [5], the major variant surface antigen on the surface of *P. falciparum* infected erythrocytes that binds chondroitin sulfate A (CSA) [6] to enable sequestration in the placenta [7], increase with parity [8]. These antibodies have been associated with reduced risk of placental infection [5, 9–11], low birth weight [12–15] and maternal anaemia [14, 16]. However, several other studies have failed to show such a protective association [11, 17–22], but instead suggested that antibodies at delivery reflect exposure to *P. falciparum* in pregnancy [8, 9, 21–28].

Recent studies show how the power to identify immune correlates of protection in children [29–31] and pregnant women [28] under heterogeneous intensities of malaria transmission can be reduced by inclusion of individuals with different degrees of exposure [29]. Heterogeneity in exposure to *P. falciparum* [9, 22, 27] can be taken into account by including in the analysis only women with proven parasite exposure before delivery (i.e., having had a malaria episode during pregnancy) [28]. However, this requires the morbidity surveillance at antenatal care units of rural hospitals in African countries that is not always available. There is a need to develop better methods to adjust for heterogeneity in parasite exposure when assessing immune responses that contribute to protection against malaria infection in pregnancy [30]. The aim of this study was to assess an alternative way of accounting for the effect of heterogeneous exposure to *P. falciparum* in the absence of information before delivery. Specifically, the objective was to assess the relationship of IgG and IgM against *P. falciparum* antigens in Mozambican pregnant women at delivery with parasite infection and adverse clinical impacts in the mothers and newborns. Pregnancy-specific antigens (infected erythrocytes selected from binding to CSA and placental isolates), general (non-pregnancy specific) antigens (merozoite antigens, non-CSA binder *P. falciparum* lines and isolates) and a parasite lysate (to assess responses against all whole-parasite antigens) were included. IgG but also IgM were assessed given their contribution for protection against infection [32], both in maternal and cord blood to assess the effect of placentally-transferred antibodies in reducing the risk of clinical malaria in children during their 1st year of life. The study was conducted in a mother and child cohort living in a malaria endemic area of Mozambique, in the context of a randomized, double-blind, placebo-controlled trial [“Age of exposure and immunity to malaria in infants” (AgeMal)] [33].

## Methods

### Study area

The study was conducted at the Centro de Investigação em Saúde de Manhiça (CISM), located in the Manhiça District, Maputo Province, southern Mozambique. The area has been described in detail elsewhere [34]. Transmission of *P. falciparum* is perennial and of moderate intensity with a warm rainy season from November to April, and a cool dry season during the rest of the year.

### Study design and samples collection

HIV-negative pregnant women resident in the Manhiça study area were recruited during the third trimester of pregnancy at the antenatal clinic of the Maragra Health Post (MHP), in the south of the study area, from September 2005 to March 2007 [33]. After delivery, neonates were evaluated for eligibility. Exclusion criteria included birth weight < 2 kg, twins, congenital malformations, birth asphyxia or apparent health problems. Three-hundred and 49 eligible newborns were enrolled in the trial.

At delivery, maternal peripheral and cord blood samples collected into EDTA vacutainers were centrifuged and plasma stored at − 20 °C for future antibody determinations. Thin and thick smears of peripheral and cord blood were Giemsa-stained and examined for malarial parasites according to quality-control procedures [33]. Haemoglobin (Hb) levels were determined on full blood counts performed using a Sysmex KX-21N cell counter (Sysmex Corporation, Kobe, Japan). Peripheral and cord blood was also collected onto filter papers for *P. falciparum* detection by real-time quantitative PCR (RTqPCR) in duplicates [35]. Tissue samples obtained from the maternal side of the placenta were processed for histological examination and classified as negative, acute, chronic or past infections [36]. When the delivery occurred outside the maternity post, only the mother blood sample was collected.

Children were followed up until age 24 months, with weekly active case detection from birth to age 10.5 months, and monthly home visits from 10.5 to 24 months of age. Children were examined and their parasitaemia and haematocrit determined if they presented fever (axillary temperature ≥ 37.5 °C) or their guardians referred history of fever in the preceding 24 h. Additionally, passive case detection was carried out at the MHP and Manhiça District Hospital (MDH) through the morbidity surveillance system to monitor attendances to the outpatient clinics and admissions to hospital.

Informed consent was sought to enroll pregnant women and their newborn children in the study upon birth. The protocol was approved by the National Mozambican Ethics Review Committee and the Hospital Clínic of Barcelona Ethics Review Committee. The trial was conducted according to the ICH Good Clinical Practice guidelines and reviewed by a Local Safety Monitor and a Data and Safety Monitoring Board. The trial was registered in ClinicalTrials.gov (clinical trials identifier NCT00231452).

### Measurement of antibodies against recombinant antigens

Recombinant merozoite surface protein 1 (MSP1; C-terminal 19-kD fragment, 3D7 strain) [37], erythrocyte-binding antigen 175 (EBA175; region F2, Camp strain) [38], apical membrane antigen-1 (AMA1; full ectodomain, 3D7 strain) [39], Duffy binding-like alpha domain (DBLα; CR1-binding minimal domain of the erythrocyte membrane protein 1 expressed by *P. falciparum* R29-rosetting + line) [40] were produced in *Escherichia coli*, refolded if the protein was insoluble and purified (> 95%) following previously described methods [38]. Enzyme-linked immunosorbent assay (ELISA) plates were coated with 200 ng of recombinant antigen per well and sequentially incubated with 100 μL of peripheral and cord plasma (1:500) in duplicate, and secondary peroxidase-conjugated goat anti-human IgG (1:30,000) or IgM (1:2000) [21, 41]. Reactions were developed as described previously [21] and OD values determined at 492 nm. Fifteen plasma samples from peripheral and cord blood of Spanish pregnant women who had never been exposed to malaria were used as negative controls. The ELISA reactivity of each sample was expressed as the percentage of the value obtained with the positive pool (11 plasma samples from hyperimmune Mozambican adults) in each experiment.

### Measurement of antibodies against whole parasite extract

Whole-parasite lysate was prepared by three freezing/thawing cycles of asynchronous in vitro cultures of 3D7, R29 and E8B laboratory strains at a 5% level of parasitaemia and 1% haematocrit, as previously described [21]. Non-infected erythrocyte (NIE) lysate, prepared using the same procedure as whole-parasite lysate, was used as a control of unspecific recognition for each plasma sample. ELISA plates were coated with 50 µL of lysate per well. Wells were blocked with 300 µL of 5% skim milk at 4 °C for 8 h. One hundred microliters of plasma sample were tested in duplicate for IgG and IgM (dilution, 1: 1600). Incubation of antibodies and development of the reaction were performed as described above. Malaria-specific antibody recognition was evaluated by subtracting the mean OD value of NIEs from the mean OD value of IEs. The pool of positive plasmas was used to normalize the data from different ELISAs and results were expressed as arbitrary units. Cutoff values for positivity were calculated as the arithmetic mean plus 3 standard deviations of 14 plasma samples from Spanish pregnant women who had never been exposed to malaria.

### Measurement of antibodies against the surface of *Plasmodium falciparum*-infected erythrocytes

Cryopreserved O+ erythrocytes infected by two placental isolates previously confirmed to express VAR2CSA [25] (IE_Plac1_ and IE_Plac2_), five pediatric isolates (IE_Ch1–Ch4_ and IE_SEV_) from Manhiça [25] and a CSA-binding parasite line adapted to culture (CS2) were thawed and matured to trophozoite stage without in vitro expansion. Erythrocytes at 1% haematocrit were sequentially incubated with test plasma (1:20), rabbit anti-human IgG (1:200) and AlexaFluor donkey anti-rabbit IgG (1:1000) plus 10 μg/mL of ethidium bromide. Reactivity against the surface of IEs was expressed as the difference between the geometric mean fluorescence intensity (MFI) of 1000 IEs and the MFI of NIE obtained in a FACSCalibur flow cytometer (BD, San Jose, USA). A pool of plasma samples from 10 Mozambican pregnant women was used to normalize the data from different assays [21] and results were expressed as arbitrary units.

### Definitions and statistical methods

Pregnant women were classified into first-time mothers (primigravida, PG), those with one previous pregnancy (secundigravia, SG) and those with more than two previous pregnancies (multigravida, MG). Age was categorized as ≤ 20, 21–24 and ≥ 25 years. Maternal anaemia was considered if the haemoglobin level was < 11 g/dL and low birth weight if the newborn weight was < 2500 g. Distance to the river was calculated with Hawth’s tools in ArcGIS software (ESRI) as the mean distance of each neighbourhood centroid to the Incomati river, and neighbourhoods were classified as those adjacent to the river (≤ 2.5 km) or those at a higher distance (> 2.5 km) [28]. Season during pregnancy was defined as dry if the period of pregnancy included the cool dry season (May to October), and rainy if it included four or more rainy months (November to April). Maternal malaria in peripheral blood was defined as the presence of parasites detected in peripheral blood by optical microscopy and/or RTqPCR. Placental malaria was defined by the presence of malaria parasites and/or pigment as detected by histology. Placental inflammation was defined as the presence of > 5 mononuclear and/or polymorphonuclear leukocytes in the intervillous spaces, assessed in 10 high power fields at 400× [42].

Continuous variables were compared by ANOVA, and categorical variables by Chi squared test or Fisher’s exact test. To identify factors that affect maternal IgG and IgM against *P. falciparum* antigens, the association of antibody levels (log transformed ODs or MFIs) with *P. falciparum* infection (maternal, placental, cord), placental inflammation, parity, neighbourhood, season and use of insecticide treated nets (ITN) or indoor residual spraying (IRS) were assessed by linear regression models. For those variables with negative values (IgGs against infected erythrocytes), prior to applying the log transform a constant value was added to the data to obtain a minimum value less than 1. All the analyses were done univariate and multivariate, adjusting for all the other independent variables. To determine the relationship of antibody responses with clinical outcomes, parasite infection and clinical outcomes in mothers and their children, the association of antibody levels (independent variable) with maternal haematocrit and weight of the newborn (dependent variable) was assessed, including a multivariate analysis with all the antibody responses as independent variables. Negative binomial regression models were used to evaluate the effect of doubling the levels of antibodies on the incidence of multiple malaria episodes in children up to 12 months of age [33]. Analyses were adjusted for maternal age, parity, use of IRS, use of ITN, season, neighbourhood, child anti-malarial intervention [33] and *P. falciparum* infection in the mother. To assess if parity modified the associations, an interaction term was included in the regression models, and ratios and 95% confidence intervals (95% CI) for each antibody responses were estimated after stratifying the regression models. Analyses were performed using Stata 11 (College Station, TX, USA) and significance was defined at p < 0.05.

## Results

### Factors related to *P. falciparum* infection in pregnancy

The analysis of antibody responses included 207 women (Table 1), 42 (20%) with peripheral infection at delivery, 47 (23%) with placental infection (acute infection in 5 [11%], chronic infection in 1 [2%] and past infection in 41 [87%]), and 8 (4%) with cord blood infection. Placental inflammation in 10 (5%) of the women was more frequent in women with peripheral infection than uninfected women. Placental infection decreased with parity (Table 1) and age of the women (35% [25 out of 71] if ≤ 20 years, 16% [9 out of 58] if 21–24 years and 17% [13 out of 78] if ≥ 25 years; p = 0.011), and was more frequent among women living in a house that had not received IRS (29% [32 out of 109]) than in those living in a house that received IRS (15% [15 out of 98]; p = 0.020). Cord blood infection was more frequent among women with placental inflammation (20% [2 out of 10]) than in those without inflammation (3% [6 out of 197]; p = 0.05). The use of ITN was higher among MG women (17% [18 out of 103]) than SG (4% [2 out of 47]) or PG women (5% [3 out of 57]; p = 0.020). LBW was more frequent among PG women than SG or MG women (Table 1).

**Table 1:** Demographic and clinical factors of mothers at delivery according to their parity

	All (n = 207)	Primigravida (n = 57)	Secundigravida (n = 47)	Multigravida (n = 103)	p
Placental infection, n (%)	47^a^ (23)	21 (37)	9 (19)	17 (17)	0.013
Peripheral infection, n (%)	42 (20)	14 (25)	7 (15)	21 (20)	0.473
Cord infection, n (%)	8 (4)	2 (4)	1 (2)	5 (5)	0.895
Placental density	90.5 (83.9–97.6)	81.1 (63.9–102.8)	98.55 (97.7–99.5)	92.6 (85.6–100.1)	0.166
Peripheral density					
Microscopy	3988 (894–17,778)	6663 (137–323,311)	3652	3058 (507–18,439)	0.897
PCR	0.69 (0.25–1.88)	1.05 (0.15–7.36)	0.81 (0.02–33.5)	0.47 (0.12–2.04)	0.793
Inflammation, n (%)	10 (5)	5 (9)	1 (2)	4 (4)	0.270
Age, mean (SD)					
15–20 years	71 (34)	44 (77)	24 (51)	3 (3)	< 0.001
21–24 years	58 (28)	12 (21)	20 (43)	26 (25)	
≥ 25 years	78 (38)	1 (2)	3 (6)	74 (72)	
Neighbourhood (< 2.5 km), n (%)	41 (20)	14 (25)	7 (30)	20 (19)	0.494
Use of ITN, n (%)	23 (11)	3 (5)	2 (4)	18 (17)	0.002
Household IRS, n (%)	98 (47)	24 (42)	21 (45)	53 (51)	0.500
Season (dry), n (%)	58 (28)	18 (32)	16 (34)	24 (23)	0.295
Low-birth weight, n (%)	28 (14)	15 (26)	6 (13)	7 (7)	0.004
Maternal anaemia, n (%)	87 (42)	23 (40)	20 (43)	44 (43)	0.792

*SD* standard deviation, *ITN* insecticide-treated nets, *IRS* indoor residual spraying

^a^41 past, 5 acute and 1 chronic infection

### Factors related to antibody levels at delivery

Placental infection was associated with higher levels of maternal and cord IgGs against all the antigens measured (except IE_SEV_), as well as with maternal IgMs against MSP1, EBA175, AMA1 and parasite lysate (Additional file 1 Table S1). Peripheral infection (adjusted for placental infection) was associated with increased IgG levels against EBA175, parasite lysate, DBLα and the two placental isolates, as well as cord IgGs against DBLα (Additional file 1 Table S1).

Maternal IgM against MSP1, EBA175, AMA1 and DBLα were lower in women with placental inflammation compared to women without inflammation (Table 2). IgM_AMA1_ was higher in *P. falciparum* positive cord blood (10.87, SD 9.40) than in negative cords (6.82, SD 3.75, p = 0.023).

**Table 2:** Antibody responses in women with and without placental infection

	No inflammation (n = 197)		Inflammation (n = 10)		p	Multivariate^a^		
	GM	SD	GM	SD		Ratio	(95% CI)	p
Maternal IgGs								
MSP1	65.41	58.42	62.84	62.76	0.891	0.88	(0.49; 1.57)	0.665
EBA175	44.17	33.09	42.24	26.13	0.853	0.89	(0.55; 1.44)	0.636
AMA1	61.45	37.81	63.18	30.41	0.889	0.96	(0.64; 1.42)	0.824
DBLa	77.56	30.62	69.28	21.26	0.375	0.83	(0.65; 1.07)	0.145
Lysate	31.68	40.32	43.07	26.07	0.450	1.16	(0.52; 2.58)	0.719
CS2	2.16	1.62	1.83	1.07	0.492	0.93	(0.79; 1.10)	0.396
Women1	149.75	227.78	93.63	96.67	0.336	0.68	(0.28; 1.62)	0.386
Women2	412.18	580.46	216.39	228.31	0.156	0.56	(0.24; 1.28)	0.171
SEV	2.72	0.88	2.39	0.90	0.228	0.97	(0.93; 1.02)	0.245
Child1	249.09	227.92	178.53	187.45	0.266	0.67	(0.37; 1.22)	0.193
Child2	641.87	547.08	544.20	448.88	0.550	0.82	(0.47; 1.44)	0.496
Child3	653.83	474.55	443.80	366.64	0.103	0.67	(0.42; 1.08)	0.105
Child4	183.91	180.67	128.46	158.73	0.267	0.64	(0.34; 1.23)	0.186
Maternal IgMs								
MSP1	75.22	45.36	40.66	32.52	0.002	0.53	(0.36; 0.80)	0.002
EBA175	39.44	27.08	18.37	15.24	0.001	0.46	(0.29; 0.72)	0.001
AMA1	51.87	31.25	29.61	28.32	0.006	0.58	(0.38; 0.86)	0.008
DBLa	76.29	38.64	39.61	35.11	< 0.001	0.53	(0.38; 0.74)	< 0.001
Lysate	16.06	29.12	15.58	24.48	0.958	0.91	(0.28; 2.93)	0.87
Cord IgGs								
MSP1	54.18	51.79	41.54	45.09	0.396	0.69	(0.37; 1.28)	0.237
EBA175	40.16	31.79	40.62	33.98	0.965	0.92	(0.55; 1.55)	0.753
AMA1	60.89	40.77	67.26	27.17	0.643	1	(0.66; 1.53)	0.989
DBLa	60.72	27.78	65.68	28.77	0.597	0.98	(0.73; 1.31)	0.891
Lysate	28.55	34.11	25.50	17.50	0.768	0.77	(0.36; 1.64)	0.495
CS2	2.30	1.73	2.12	1.39	0.726	0.98	(0.83; 1.16)	0.804
SEV	2.61	0.81	2.35	0.74	0.304	0.98	(0.94; 1.02)	0.288
Cord IgMs								
MSP1	10.76	5.54	11.92	8.08	0.546	1.1	(0.79; 1.54)	0.575
EBA175	4.83	3.04	6.36	6.91	0.197	1.3	(0.85; 1.99)	0.231
AMA1	6.88	3.79	8.43	7.34	0.272	1.23	(0.85; 1.77)	0.275
DBLa	10.67	5.45	12.57	9.02	0.335	1.18	(0.84; 1.64)	0.344
Lysate	6.89	5.64	6.85	5.77	0.984	1.06	(0.62; 1.81)	0.827

*GM* geometric mean, *SD* standard deviation

^a^Adjusted for parity, age, neighbourhood, ITN, season and IRS

The use of ITNs was associated with reduced levels of maternal IgGs against DBLα (Additional file 1 Table S1), while IRS was associated with a reduction in the levels of maternal IgGs against lysate (Additional file 1 Table S1). Antibodies against IE_ch3_ were 1.32-fold higher in neighbourhoods close to the river (< 2.5 km) compared to those at more than < 2.5 km (95% CI [1.02; 1.69]; p = 0.037). There were no significant differences according to season (all p values > 0.05).

**Fig. 1:**
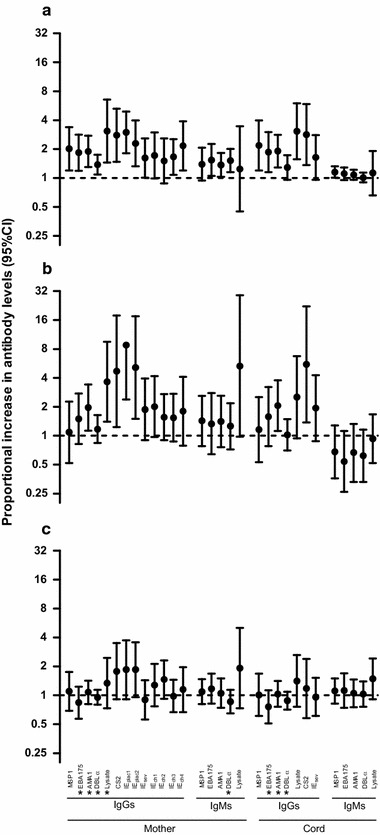
Association between placental infection and antibody levels by parity. Multivariate regression analysis adjusted by peripheral infection, age, neighbourhood, household indoor residual spraying, use of insecticide treated nets and season. Y axis represents the proportional increase in the level of antibodies in women with a placental infection compared to uninfected women among primigravida (**a**), secundigravida (**b**) and multigravida (**c**). *If the p value for the interaction term between parity and antibody levels was < 0.05

Parity modified the association of maternal IgG and IgM responses as well as cord IgGs with placental infection, as indicated by the statistically significant interaction terms (Fig. 1). The analysis stratified by parity and infection showed that the increase of antibody responses associated with placental infection was mainly observed in PG women (Fig. 1).

### Factors related to pregnancy outcome and incidence of malaria in the children

The newborn weight increased with age and parity of the women, and was lower among women with a placental infection (Table 3). Haemoglobin levels were lower among women who had their pregnancy during the rainy season. Incidence of malaria during the first 1 year of life was higher among children born from a mother with a cord blood infection (Table 3).

**Table 3:** Factors associated with newborn weight, maternal haemoglobin levels and incidence of malaria during the 1st year of life

	Newborn weight (kg)			Haemoglobin (g/L)			Incidence malaria children		
	Mean	95% CI	p	Mean	95% CI	p	IRR	95% CI	p^b^
Parity									
PG	2.8	2.69–2.91	< 0.001	113.1	105.4–120.7	0.878	1		
SG	2.9	2.81–2.99		113.3	107.9–119.6		1.64	0.61–4.43	0.331
MG	3.13	3.05–3.20		111.5	107.2–115.8		1.07	0.44–2.59	0.878
Placental infection									
Negative	3.02	2.95–3.08	0.044	113	109.4–116.6	0.473	1		
Positive	2.89	2.77–2.99		110.1	102.1–118.1		0.95	0.39–2.27	0.915
Peripheral infection									
Negative	2.99	2.92–3.05	0.990	113.3	109.6–116.9	0.288	1		
Positive	2.99	2.89–3.08		108.9	101.1–116.7		1.82	0.79–4.18	0.161
Inflammation									
Negative	2.98	2.92–3.03	0.106	112.6	109.3–116.0	0.488	1		
Positive	3.19	2.92–3.46		107.0	90.26–123.7		2.96	0.72–12.02	0.130
Cord infection									
Negative	2.98	2.92–3.03	0.131	112.6	109.3–115.9	0.474	1		
Positive	3.2	2.72–3.68		106.4	88.9–123.9		5.90	1.53–22.77	0.010^a^
Age (years)									
15–20	2.78	2.69–2.87	< 0.001^a^	110.7	104.7–116.8	0.344	1		
21–24	3.03	2.95–3.12		116.2	110.0–122.3		0.79	0.31–1.98	0.608
≥ 25	3.14	3.05–3.23		110.9	105.7–116.0		0.88	0.38–2.05	0.769
Neighbourhood (km)									
> 2.5	3.00	2.94–3.06	0.328	111.1	114.7–107.5	0.122	1		
< 2.5	2.93	2.79–3.07		117.6	109.9–125.2		1.04	0.42–2.57	0.936
Use of ITN									
Negative	2.97	2.91–3.03	0.171	112.8	109.2–116.5	0.469	1		
Positive	3.09	2.92–3.28		109.1	102.4–115.9		1.07	0.34–3.35	0.903
Use of IRS									
Negative	2.94	2.87–3.02	0.109	113.3	108.5–118.1	0.573	1		
Positive	3.04	2.95–3.12		111.4	106.8–115.9		0.66	0.32–1.37	0.268
Season									
Rainy	2.98	2.91–3.05	0.685	110.2	106.2–114.3	0.049^a^	1		
Dry	3.01	2.89–3.11		117.4	111.8–123.0		0.99	0.44–2.21	0.980

*PG* primigravida, *SG* secundigravida, *MG* multigravida, *ITN* insecticide-treated nets, *IRS* indoor residual spraying, *IRR* incidence rate ratio

^a^p < 0.05 in the multivariate analysis adjusted by age, use of IRS and ITS, season and neighbourhood

^b^adjusted for treatment, weight and maternal haemoglobin

**Fig. 2:**
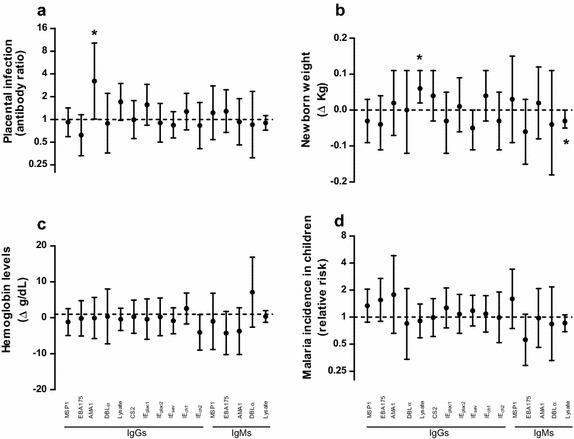
Association of antibody levels with maternal infection, pregnancy outcomes and incidence of malaria in children. Multivariate analysis including all the antibodies and adjusted for parity, age, neighbourhood, season, household indoor residual spraying and use of insecticide treated nets. Y axis represent the adjusted ratio of antibody levels between women with placental infection and women without infection (**a**), the effect of a twofold increase in antibody levels on the newborn weight (**b**), on maternal haemoglobin levels (**c**) and on the risk of malaria episodes in infants (**d**). Δ Expresses the increase or decrease of newborn weight or maternal haemoglobin levels with twofold increases in antibody levels. * identifies statistically significant p values (< 0.05)

The newborn weight decreased with increasing levels of maternal IgM_EBA175_ and IgM_DBLα_ (reductions of 0.06 kg (95% CI [(− 0.11; − 0.01], p = 0.031 and 0.07 kg (95% CI [− 0.14; − 0.01], p = 0.032) with twofold increase in antibody levels, respectively; Additional file 1: Table S2). No association was found between antibody levels and maternal anaemia. The risk of malaria in the children during their 1st year of life increased with increasing levels of maternal IgGs (all except IgG_ch1_) and cord IgGs (all except IgG-IE_Sev_). The multivariate analysis including all antibody levels showed that newborn weight increased with levels of IgG_lysate_ whereas decreased with levels of IgM_lysate_ (Fig. 2; Additional file 1: Table S3).

## Discussion

This study shows that placental infection is associated with increased levels of maternal IgG and IgM against a broad range of parasite antigens and that the newborn weight decreases with increasing levels of IgM specific for EBA175 and DBLα. No association was found between IgG levels against any of the antigens tested and improved pregnancy outcomes when the analysis was performed for specific antibodies. However, higher levels of IgG against a parasite lysate were associated with increasing birth weight when IgG and IgM were included in a multivariate analysis, being IgM against the parasite lysate associated with a reduction in the newborn weight. Overall, these results suggest that adjusting the analysis for the simultaneous effect of IgM and IgG could contribute to adjust for heterogeneity in parasite exposure when assessing immune responses that confer protection against malaria infection in pregnancy, being IgM markers of parasite exposure and IgG markers of protection against poor pregnancy outcomes.

Results of this study confirm parasitological observations already described for malaria in pregnancy. PG and younger women (15–20 year) were at higher risk of placental infection as detected by histology [43]. This increased risk can be attributed to a higher susceptibility to malaria during first pregnancies due to associated immune modulation [25] and/or lack of immunity against VAR2CSA expressed by placental parasites [5]. Differential use of preventive tools among women at different parities, as shown by the higher use of ITN among MG women compared to PG, may also contribute to reduce the risk of malaria infection in MG women. In accordance with this reduction of placental infection with increasing parity, low birth weight was more common in PG and the newborn weight was reduced in mothers with placental infection and younger women. Importantly, household IRS was also associated with a reduction in the prevalence of placental infection. In contrast to placental infection, peripheral infection was not found to be associated with parity, possibly because placental submicroscopic infections were not assessed in this study. Results of this study also show that incidence of malaria was higher among children born from women living closer to the river, an association that can be explained by similar risk of exposure to the parasites in mothers and infants living in the same household [21]. Furthermore, this study shows that cord blood infections are associated with an increased risk of malaria during the 1st year of life, suggesting that in utero exposure can have a detrimental effect on the ability of newborns to mount an effective immune response against the parasite.

In this maternal cohort, placental infection is associated with an increase of IgG, as already shown in studies conducted in the same study area [21, 28], but also with increasing IgM. The magnitude of this increase was higher in PG than in MG, probably because parasite densities are higher in first time pregnant women, leading to a higher boosting of antibodies. Antibody responses were associated with a non-significant reduction of haemoglobin levels in the women at delivery, and the multivariate analysis showed that IgM against the lysate were associated with a reduction in the newborn weight. Although the large number of comparisons made may have increased the chance of false discoveries, the fact that this associations with antibody responses against parasite lysate are maintained in the multivariate analysis including all the antigens suggest the genuineness of the observation. Moreover, antibody responses were associated with an increase in the incidence of malaria in their children during their 1st year of life. This association may be explained by a similarly high exposure to mosquito bites in pregnant women and their children residing in the same household which would translate into a boosting of antibody responses in pregnant women at delivery and a higher malaria incidence in the children. Overall, these results reinforce the concept of antibodies as markers of parasite exposure and the difficulties of disentangling the protective versus exposure role of antibodies in a situation of heterogeneous exposure. In absence of a continuous morbidity surveillance at antenatal care units to identify malaria cases and exclude from the analysis those women without proven exposure during pregnancy (i.e., having had a malaria episode), these results suggest that adjusting for IgM as markers of exposure to the parasite can control for the heterogeneity of exposure [28] and contribute to identify those IgG that may have a role in protection. Interestingly, data of this study show that IgG against a parasite lysate are more associated to an increase in the newborn weight than IgG against the surface of a CSA-binding parasite line. This association might be explained by an antibody-mediated clearance of circulating parasites early at pregnancy (i.e., before these parasite populations switch to placental-binding, that can contribute to prevent low birthweight. Also, immunity against a broad range of *P. falciparum* antigens and not only against CS2 may have a role in controlling parasite infections and reducing the adverse consequences of malaria in pregnancy.

## Conclusions

Newborn weight increased with increasing IgG levels against a parasite lysate, whereas the opposite association was found with IgM, suggesting that IgG are markers of protection against poor pregnancy outcomes and IgM of parasite exposure. This study confirms the need for developing methods to control for heterogeneous exposure to the parasite when aiming to identify those immune responses that have a potential role in protection against infection and disease. Further studies are needed to assess novel approaches to disentangle exposure from protection in immunological studies assessing the role of antibodies against the adverse consequences of malaria infection in situations of heterogeneous malaria exposure.

## Additional file

**Additional file 1: Table S1.** Association of antibody responses with maternal infection, use of insecticide treated nets and indoor residual spraying. **Table S2.** Association between antibody levels and weight of newborn, maternal hemoglobin and incidence of malaria in children. **Table S3.** Association of antibody levels with maternal infection, pregnancy outcomes and incidence of malaria in children.

## Abbreviations

MSP1: merozoite surface protein 1
EBA175: erythrocyte-binding antigen 175
AMA1: apical membrane antigen-1
DBLα: Duffy binding-like alpha domain
ELISA: enzyme-linked immunosorbent assay
IgG: immunoglobulin G
IgM: immunoglobulin M
IE: infected erythrocytes
CSA: chondroitin sulfate A
RTqPCR: real-time quantitative PCR
Hb: haemoglobin
NIE: non-infected erythrocyte
MFI: mean fluorescence intensity
CISM: Centro de Investigação em Saúde de Manhiça
MDH: Manhiça District Hospital
MHP: Maragra Health Post
HIV: human immunodeficiency virus
PG: primigravida
SG: secundigravida
MG: multigravida
ITN: insecticide-treated nets
IRS: indoor residual spraying
