# Supplementary material for: IgM and IgG against Plasmodium falciparum lysate as surrogates of malaria exposure and protection during pregnancy
Source: Malar J. 2018 May 10;17:182. doi: 10.1186/s12936-018-2331-4 (PMC5944166; doi:10.1186/s12936-018-2331-4)
Supplement: Supplementary file 1 — Additional file 1: Table S1. Association of antibody responses with maternal infection, use of insecticide treated nets and indoor residual spraying. Table S2. Association between antibody levels and weight of newborn, maternal hemoglobin and incidence of malaria in children. Table S3. Association of antibody levels with maternal infection, pregnancy outcomes and incidence of malaria in children. [file 12936_2018_2331_MOESM1_ESM.docx]

**Table S1. Association of antibody responses with maternal infection, use of insecticide treated nets and indoor residual spraying.**

|  |  | **Placental infection** | | | |  |  | **Peripheral infection** | | | |  |  | **Use of ITNs** | | | |  |  | **Household IRS** | | | |  |
| --- | --- | --- | --- | --- | --- | --- | --- | --- | --- | --- | --- | --- | --- | --- | --- | --- | --- | --- | --- | --- | --- | --- | --- | --- |
|  |  | **Neg (n=160)** | | **Pos (n=47)** | |  |  | **Neg (n=165)** | | **Pos (n=42)** | |  |  | **No (n=184)** | | **Yes (n=23)** | |  |  | **No (n=109)** | | **Yes (n=98)** | |  |
|  |  | **GM** | **SD** | **GM** | **SD** | **p** |  | **GM** | **SD** | **GM** | **SD** | **p** |  | **GM** | **SD** | **GM** | **SD** | **p** |  | **GM** | **SD** | **GM** | **SD** | **p** |
| **Maternal IgGs** | |  |  |  |  |  |  |  |  |  |  |  |  |  |  |  |  |  |  |  |  |  |  |  |
|  | **MSP1** | 59.3 | 52.8 | 90.4 | 76.5 | **0.004*** |  | 60.0 | 53.0 | 91.1 | 79.8 | **0.007** |  | 66.5 | 59.7 | 56.1 | 49.6 | 0.392 |  | 70.0 | 68.1 | 60.4 | 48.3 | 0.235 |
|  | **EBA175** | 40.8 | 29.9 | 57.4 | 41.5 | **0.005*** |  | 40.4 | 30.1 | 61.9 | 39.1 | **0.001*** |  | 44.8 | 33.5 | 38.7 | 27.3 | 0.371 |  | 46.2 | 34.3 | 41.8 | 31.0 | 0.332 |
|  | **AMA1** | 56.3 | 36.5 | 83.3 | 23.9 | **0.000*** |  | 59.1 | 38.3 | 72.4 | 27.7 | 0.053 |  | 62.5 | 36.9 | 54.3 | 40.4 | 0.299 |  | 65.3 | 37.4 | 57.6 | 37.0 | 0.141 |
|  | **DBLα** | 73.4 | 25.8 | 91.4 | 42.9 | **0.001*** |  | 73.0 | 27.1 | 95.7 | 38.2 | **0.000*** |  | 79.0 | 31.2 | 64.0 | 19.3 | **0.015*** |  | 80.0 | 34.7 | 74.0 | 24.8 | 0.152 |
|  | **Lysate** | 26.1 | 34.1 | 65.5 | 42.6 | **0.000*** |  | 27.4 | 35.4 | 59.9 | 49.7 | **0.000*** |  | 33.2 | 41.5 | 24.6 | 31.0 | 0.279 |  | 39.1 | 41.8 | 25.9 | 36.2 | **0.018*** |
|  | **CS2** | 2.0 | 1.5 | 2.9 | 1.8 | **0.002*** |  | 2.0 | 1.5 | 2.7 | 1.9 | 0.017 |  | 2.1 | 1.6 | 2.3 | 1.4 | 0.575 |  | 2.2 | 1.7 | 2.0 | 1.5 | 0.354 |
|  | **IE_plac1_** | 119.4 | 178.3 | 297.5 | 392.9 | **0.000*** |  | 123.5 | 182.3 | 284.3 | 407.6 | **0.001*** |  | 143.2 | 217.9 | 174.4 | 237.2 | 0.555 |  | 161.1 | 236.5 | 131.7 | 202.9 | 0.339 |
|  | **IE_plac2_** | 330.6 | 454.9 | 764.3 | 983.1 | **0.000*** |  | 335.6 | 453.7 | 781.5 | 1082.1 | **0.000*** |  | 394.9 | 563.3 | 436.6 | 512.0 | 0.746 |  | 424.0 | 592.6 | 373.8 | 524.3 | 0.522 |
|  | **IE_SEV_** | 2.6 | 0.8 | 2.9 | 1.0 | 0.079 |  | 2.7 | 0.7 | 2.8 | 1.3 | 0.395 |  | 2.7 | 0.9 | 2.5 | 0.8 | 0.235 |  | 2.7 | 0.8 | 2.6 | 0.9 | 0.434 |
|  | **IE_ch1_** | 220.2 | 203.8 | 356.0 | 289.2 | **0.002*** |  | 227.6 | 201.9 | 327.4 | 329.5 | **0.022** |  | 239.2 | 219.0 | 297.3 | 288.9 | 0.288 |  | 269.2 | 231.7 | 221.1 | 216.4 | 0.126 |
|  | **IE_ch2_** | 589.1 | 510.0 | 833.1 | 616.0 | **0.014*** |  | 629.1 | 490.1 | 667.1 | 728.4 | 0.691 |  | 633.8 | 545.9 | 660.5 | 507.5 | 0.827 |  | 670.1 | 585.6 | 602.2 | 495.9 | 0.370 |
|  | **IE_ch3_** | 607.3 | 479.9 | 774.5 | 348.0 | **0.048** |  | 615.7 | 463.8 | 751.7 | 477.4 | 0.116 |  | 650.7 | 466.1 | 574.1 | 497.9 | 0.442 |  | 670.5 | 455.5 | 611.1 | 482.6 | 0.368 |
|  | **IE_ch4_** | 162.6 | 167.3 | 259.8 | 202.0 | **0.005*** |  | 168.7 | 167.2 | 235.5 | 231.0 | 0.053 |  | 177.9 | 177.1 | 204.0 | 206.2 | 0.536 |  | 202.1 | 190.6 | 159.7 | 166.4 | 0.092 |
| **Maternal IgMs** | |  |  |  |  |  |  |  |  |  |  |  |  |  |  |  |  |  |  |  |  |  |  |  |
|  | **MSP1** | 69.0 | 38.6 | 88.5 | 69.9 | **0.016*** |  | 72.7 | 43.7 | 74.3 | 53.6 | 0.840 |  | 72.6 | 46.8 | 76.8 | 34.9 | 0.681 |  | 71.4 | 48.2 | 74.8 | 42.5 | 0.595 |
|  | **EBA175** | 35.3 | 23.4 | 48.9 | 39.9 | **0.006*** |  | 36.0 | 24.7 | 46.9 | 36.4 | **0.031** |  | 38.2 | 27.4 | 36.7 | 24.7 | 0.804 |  | 37.5 | 28.0 | 38.6 | 26.0 | 0.761 |
|  | **AMA1** | 47.9 | 27.6 | 60.3 | 46.7 | **0.028*** |  | 48.3 | 29.4 | 60.1 | 42.1 | **0.046** |  | 50.2 | 32.7 | 53.1 | 24.3 | 0.682 |  | 49.6 | 32.7 | 51.5 | 31.0 | 0.662 |
|  | **DBLα** | 71.8 | 36.3 | 81.8 | 54.0 | 0.150 |  | 74.0 | 39.5 | 73.5 | 44.1 | 0.939 |  | 72.6 | 40.8 | 85.1 | 32.0 | 0.190 |  | 72.4 | 42.4 | 75.7 | 37.9 | 0.557 |
|  | **Lysate** | 13.6 | 25.1 | 28.5 | 42.1 | **0.013*** |  | 14.0 | 25.8 | 27.7 | 40.8 | **0.027** |  | 15.8 | 28.7 | 17.8 | 30.9 | 0.765 |  | 16.4 | 29.0 | 15.7 | 28.8 | 0.857 |
| **Cord IgGs** | |  |  |  |  |  |  |  |  |  |  |  |  |  |  |  |  |  |  |  |  |  |  |  |
|  | **MSP1** | 48.8 | 46.6 | 73.2 | 68.0 | **0.012*** |  | 50.3 | 47.7 | 68.1 | 67.7 | 0.076 |  | 54.1 | 52.2 | 48.0 | 45.8 | 0.589 |  | 58.9 | 62.0 | 48.0 | 40.2 | 0.133 |
|  | **EBA175** | 37.2 | 29.5 | 52.2 | 38.5 | **0.011*** |  | 37.9 | 30.3 | 50.7 | 36.7 | **0.038** |  | 40.7 | 32.6 | 36.3 | 25.7 | 0.534 |  | 42.7 | 34.5 | 37.6 | 28.9 | 0.257 |
|  | **AMA1** | 55.8 | 39.3 | 84.0 | 25.4 | **0.000*** |  | 58.7 | 41.5 | 72.4 | 27.1 | 0.072 |  | 62.3 | 39.8 | 52.7 | 42.4 | 0.273 |  | 66.1 | 40.3 | 56.1 | 39.4 | 0.077 |
|  | **DBLα** | 58.9 | 24.5 | 68.7 | 38.7 | **0.046** |  | 58.5 | 25.5 | 72.1 | 35.8 | **0.009*** |  | 62.4 | 28.6 | 50.3 | 19.7 | **0.040** |  | 64.5 | 32.7 | 57.2 | 21.9 | 0.066 |
|  | **Lysate** | 23.4 | 28.9 | 55.4 | 30.8 | **0.000*** |  | 25.4 | 31.0 | 44.2 | 37.7 | **0.007** |  | 28.7 | 34.0 | 25.7 | 28.5 | 0.685 |  | 33.0 | 38.0 | 24.0 | 28.4 | 0.055 |
|  | **CS2** | 2.2 | 1.7 | 2.9 | 1.7 | **0.023*** |  | 2.2 | 1.7 | 2.5 | 1.8 | 0.409 |  | 2.3 | 1.7 | 2.5 | 1.5 | 0.55 |  | 2.4 | 1.7 | 2.2 | 1.7 | 0.477 |
|  | **IE_SEV_** | 2.5 | 0.7 | 2.8 | 1.0 | 0.075 |  | 2.6 | 0.7 | 2.7 | 1.0 | 0.426 |  | 2.6 | 0.8 | 2.3 | 0.7 | 0.066 |  | 2.6 | 0.8 | 2.6 | 0.8 | 0.764 |
| **Cord IgMs** | |  |  |  |  |  |  |  |  |  |  |  |  |  |  |  |  |  |  |  |  |  |  |  |
|  | **MSP1** | 10.7 | 6.1 | 11.1 | 3.5 | 0.700 |  | 10.7 | 5.9 | 11.3 | 4.4 | 0.573 |  | 10.9 | 6.0 | 9.8 | 2.3 | 0.375 |  | 11.2 | 6.4 | 10.4 | 4.8 | 0.310 |
|  | **EBA175** | 4.9 | 3.4 | 4.9 | 2.8 | 0.991 |  | 4.8 | 3.4 | 5.1 | 2.2 | 0.621 |  | 5.0 | 3.4 | 4.1 | 1.0 | 0.164 |  | 5.1 | 3.6 | 4.7 | 2.8 | 0.355 |
|  | **AMA1** | 7.0 | 4.3 | 6.7 | 2.6 | 0.648 |  | 7.0 | 4.2 | 6.7 | 2.7 | 0.625 |  | 7.0 | 4.2 | 6.2 | 1.3 | 0.345 |  | 7.1 | 4.3 | 6.8 | 3.5 | 0.638 |
|  | **DBLα** | 10.9 | 6.1 | 10.2 | 3.6 | 0.416 |  | 10.8 | 6.0 | 10.7 | 4.0 | 0.938 |  | 10.9 | 5.9 | 10.0 | 1.9 | 0.469 |  | 11.1 | 6.3 | 10.4 | 4.8 | 0.370 |
|  | **Lysate** | 6.7 | 5.8 | 7.5 | 4.8 | 0.402 |  | 7.1 | 5.6 | 6.2 | 5.7 | 0.377 |  | 6.8 | 5.7 | 7.3 | 4.8 | 0.734 |  | 6.6 | 5.5 | 7.2 | 5.8 | 0.410 |
| *, p<0.05 after adjusting for variables in the table, parity and age | | | | | | | | |  |  |  |  |  |  |  |  |  |  |  |  |  |  |  |  |
| Use of ITN, Insecticide treated nets; IRS, Indoor residual spraying; GM, Geometric mean; SD, Standard deviation | | | | | | | | | | | | | | |  |  |  |  |  |  |  |  |  |  |

## Table S2. Association between antibody levels and weight of newborn, maternal hemoglobin and incidence of malaria in children.

|  |  | **Newborn weight** | | |  | **Maternal hemoglobin** | | |  | **Incidence malaria children** | | |
| --- | --- | --- | --- | --- | --- | --- | --- | --- | --- | --- | --- | --- |
|  |  | **aEffect** | **(95%CI)** | **p** |  | **aEffect** | **(95%CI)** | **p** |  | **IRR** | **(95%CI)** | **p** |
| **Maternal IgGs** | |  |  |  |  |  |  |  |  |  |  |  |
|  | **MSP1** | -0.02 | (-0.06; 0.02) | 0.284 |  | -2.32 | (-4.91; 0.27) | 0.082 |  | 1.48 | ( 1.12; 1.93) | **0.005** |
|  | **EBA175** | -0.02 | (-0.07; 0.03) | 0.441 |  | -2.14 | (-5.28; 0.99) | 0.182 |  | 1.67 | ( 1.17; 2.37) | **0.005** |
|  | **AMA1** | 0.02 | (-0.04; 0.08) | 0.461 |  | -2.67 | (-6.34; 0.99) | 0.155 |  | 2.88 | ( 1.39; 5.95) | **0.004** |
|  | **DBLα** | -0.04 | (-0.13; 0.06) | 0.419 |  | -2.5 | (-8.50; 3.50) | 0.416 |  | 2.02 | ( 1.08; 3.80) | **0.028** |
|  | **Lysate** | 0.02 | (-0.01; 0.05) | 0.201 |  | -1.35 | (-3.23; 0.54) | 0.163 |  | 1.39 | ( 1.07; 1.82) | **0.014** |
|  | **CS2** | 0.01 | (-0.02; 0.03) | 0.514 |  | -0.97 | (-2.63; 0.69) | 0.252 |  | 1.39 | ( 1.16; 1.66) | **<0.001** |
|  | **SEV** | -0.02 | (-0.06; 0.02) | 0.415 |  | -1.24 | (-3.88; 1.39) | 0.356 |  | 1.49 | ( 1.10; 2.01) | **0.009** |
|  | **IE_plac1_** | 0.01 | (-0.02; 0.03) | 0.667 |  | -1.14 | (-2.88; 0.61) | 0.203 |  | 1.41 | ( 1.18; 1.68) | **<0.001** |
|  | **IE_plac2_** | 0.01 | (-0.02; 0.04) | 0.461 |  | -1.15 | (-2.96; 0.66) | 0.215 |  | 1.4 | ( 1.16; 1.70) | **0.001** |
|  | **IE_SEV_** | 0.02 | (-0.02; 0.05) | 0.443 |  | -0.63 | (-3.11; 1.84) | 0.616 |  | 1.62 | ( 1.20; 2.21) | **0.002** |
|  | **IE_ch1_** | 0.01 | (-0.04; 0.05) | 0.806 |  | -1.07 | (-3.75; 1.62) | 0.437 |  | 1.08 | ( 0.82; 1.42) | 0.579 |
|  | **IE_ch2_** | 0 | (-0.05; 0.06) | 0.847 |  | -2.77 | (-5.89; 0.34) | 0.083 |  | 1.73 | ( 1.13; 2.66) | **0.012** |
|  | **IE_ch3_** | 0.01 | (-0.03; 0.05) | 0.585 |  | -1.44 | (-3.69; 0.81) | 0.210 |  | 1.7 | ( 1.26; 2.30) | **0.001** |
| **Maternal IgMs** | |  |  |  |  |  |  |  |  |  |  |  |
|  | **IE_ch4_** |  |  |  |  |  |  |  |  |  |  |  |
|  | **MSP1** | -0.05 | (-0.11; 0.01) | 0.087 |  | -1.84 | (-5.73; 2.04) | 0.353 |  | 1.16 | ( 0.82; 1.65) | 0.390 |
|  | **EBA175** | -0.06 | (-0.11;-0.01) | **0.031** |  | -3.35 | (-6.73; 0.02) | 0.053 |  | 1.12 | ( 0.82; 1.54) | 0.471 |
|  | **AMA1** | -0.04 | (-0.10; 0.01) | 0.134 |  | -3.37 | (-7.15; 0.41) | 0.082 |  | 1.28 | ( 0.87; 1.89) | 0.213 |
|  | **DBLα** | -0.07 | (-0.14;-0.01) | **0.032** |  | -1.03 | (-5.45; 3.39) | 0.648 |  | 1.11 | ( 0.73; 1.68) | 0.638 |
|  | **Lysate** | -0.01 | (-0.03; 0.01) | 0.222 |  | -0.17 | (-1.46; 1.11) | 0.795 |  | 1.04 | ( 0.90; 1.21) | 0.611 |
| **Cord IgGs** | |  |  |  |  |  |  |  |  |  |  |  |
|  | **MSP1** | -0.01 | (-0.05; 0.02) | 0.466 |  | -1.6 | (-4.08; 0.88) | 0.208 |  | 1.4 | ( 1.07; 1.81) | **0.013** |
|  | **EBA175** | 0 | (-0.05; 0.04) | 0.890 |  | -2.1 | (-5.09; 0.88) | 0.169 |  | 1.73 | ( 1.21; 2.47) | **0.003** |
|  | **AMA1** | 0.03 | (-0.03; 0.09) | 0.321 |  | -2.97 | (-6.43; 0.49) | 0.094 |  | 3.72 | ( 1.57; 8.80) | **0.003** |
|  | **DBLα** | 0.01 | (-0.08; 0.09) | 0.855 |  | -1.06 | (-6.28; 4.15) | 0.690 |  | 1.8 | ( 1.02; 3.21) | **0.044** |
|  | **Lysate** | 0.02 | (-0.01; 0.05) | 0.304 |  | -1.85 | (-3.88; 0.19) | 0.077 |  | 1.42 | ( 1.08; 1.88) | **0.013** |
|  | **CS2** | 0 | (-0.02; 0.03) | 0.717 |  | -0.87 | (-2.48; 0.74) | 0.293 |  | 1.2 | ( 1.01; 1.44) | **0.040** |
|  | **IE_SEV_** | -0.01 | (-0.05; 0.03) | 0.670 |  | -1.43 | (-4.08; 1.23) | 0.294 |  | 1.24 | ( 0.92; 1.66) | 0.157 |
| **Cord IgMs** | |  |  |  |  |  |  |  |  |  |  |  |
|  | **MSP1** | 0.01 | (-0.07; 0.08) | 0.860 |  | -1.5 | (-6.35; 3.35) | 0.545 |  | 1.4 | ( 0.88; 2.24) | 0.159 |
|  | **EBA175** | 0 | (-0.05; 0.06) | 0.865 |  | -1.29 | (-5.89; 3.31) | 0.583 |  | 1.27 | ( 0.88; 1.82) | 0.201 |
|  | **AMA1** | 0.01 | (-0.05; 0.08) | 0.682 |  | -1.41 | (-6.07; 3.25) | 0.555 |  | 1.29 | ( 0.84; 1.97) | 0.243 |
|  | **DBLα** | 0.02 | (-0.05; 0.09) | 0.628 |  | -0.09 | (-4.97; 4.80) | 0.973 |  | 1.29 | ( 0.81; 2.04) | 0.287 |
|  | **Lysate** | -0.02 | (-0.07; 0.02) | 0.349 |  | 0.05 | (-2.78; 2.88) | 0.971 |  | 1.17 | ( 0.85; 1.61) | 0.348 |
| Adjusted for parity, age, neighborhood, use of insecticide treated nets and indoor residual spraying. | | | | | | | | | | | |  |
| a, Effect, Adjusted effect refers to the effect of a 2-fold increase in antibody levels on the weight of the newborn and the maternal hemoglobin levels. | | | | | | | | | | | | |
| IRR, Incidence rate ratio refers to the effect of a 2-fold increase in antibody levels on risk of malaria episodes in infants. | | | | | | | | | | | | |

**Table S3. Association of antibody levels with maternal infection, pregnancy outcomes and incidence of malaria in children.**

Univariate and multivariate analysis including all the antibodies and adjusted for parity, age, neighborhood, season, household indoor residual spraying and use of insecticide treated nets. Table described the adjusted ratio of antibody levels between women with placental infection and women without infection, the effect of a 2-fold increase in antibody levels on the newborn weight, on maternal hemoglobin levels and on the risk of malaria episodes in infants.

|  |  | **Univariate** | | |  | **Multivariate** | | |
| --- | --- | --- | --- | --- | --- | --- | --- | --- |
|  | **VARIABLE** | **aRatio** | **(95%CI)** | **p** |  | **aEffect** | **(95%CI)** | **p** |
| **Placental infection** | |  |  |  |  |  |  |  |
|  | IgG MSP | 0.94 | (0.63; 1.40) | 0.761 |  | 0.92 | (0.59; 1.42) | 0.696 |
|  | IgG EBA175 | 0.74 | (0.44; 1.24) | 0.250 |  | 0.62 | (0.33; 1.15) | 0.130 |
|  | IgG AMA | 2.54 | (0.91; 7.10) | 0.076 |  | 3.21 | (1.01;10.23) | **0.049** |
|  | IgG DBLa | 1.68 | (0.78; 3.58) | 0.182 |  | 0.89 | (0.36; 2.21) | 0.801 |
|  | IgG lysate | 1.74 | (1.02; 2.98) | **0.042** |  | 1.70 | (0.97; 2.99) | 0.065 |
|  | IgG CS2 | 0.88 | (0.54; 1.42) | 0.596 |  | 0.99 | (0.56; 1.77) | 0.982 |
|  | IgG IE_plac1_ | 1.11 | (0.66; 1.90) | 0.688 |  | 1.56 | (0.84; 2.91) | 0.158 |
|  | IgG IE_plac2_ | 0.99 | (0.59; 1.66) | 0.973 |  | 0.90 | (0.50; 1.63) | 0.728 |
|  | IgG IE_SEV_ | 1.06 | (0.74; 1.51) | 0.748 |  | 0.84 | (0.57; 1.26) | 0.401 |
|  | IgG IE_ch1_ | 1.32 | (0.84; 2.10) | 0.233 |  | 1.27 | (0.73; 2.21) | 0.392 |
|  | IgG IE_ch2_ | 0.77 | (0.42; 1.40) | 0.391 |  | 0.83 | (0.41; 1.68) | 0.600 |
|  | IgM MSP | 1.15 | (0.57; 2.35) | 0.694 |  | 1.22 | (0.54; 2.77) | 0.632 |
|  | IgM EBA175 | 1.37 | (0.77; 2.45) | 0.282 |  | 1.28 | (0.67; 2.46) | 0.450 |
|  | IgM AMA | 1.13 | (0.62; 2.06) | 0.693 |  | 0.93 | (0.46; 1.88) | 0.845 |
|  | IgM DBLα | 0.61 | (0.25; 1.48) | 0.275 |  | 0.85 | (0.31; 2.35) | 0.758 |
|  | IgM lysate | 0.95 | (0.78; 1.16) | 0.643 |  | 0.90 | (0.72; 1.12) | 0.331 |
| **Newborn weight** | |  |  |  |  |  |  |  |
|  | IgG MSP | -0.03 | (-0.09; 0.03) | 0.351 |  | -0.03 | (-0.09; 0.03) | 0.290 |
|  | IgG EBA175 | -0.03 | (-0.10; 0.05) | 0.524 |  | -0.04 | (-0.11; 0.04) | 0.333 |
|  | IgG AMA | 0.02 | (-0.07; 0.12) | 0.605 |  | 0.02 | (-0.07; 0.11) | 0.620 |
|  | IgG DBLα | -0.05 | (-0.17; 0.06) | 0.385 |  | 0.00 | (-0.12; 0.11) | 0.973 |
|  | IgG lysate | 0.05 | (0.00; 0.09) | 0.051 |  | 0.06 | (0.02; 0.11) | **0.008** |
|  | IgG CS2 | 0.02 | (-0.05; 0.10) | 0.522 |  | 0.04 | (-0.03; 0.11) | 0.292 |
|  | IgG IE_plac1_ | 0.01 | (-0.07; 0.10) | 0.765 |  | -0.03 | (-0.12; 0.05) | 0.426 |
|  | IgG IE_plac2_ | 0.03 | (-0.05; 0.11) | 0.518 |  | 0.01 | (-0.06; 0.09) | 0.727 |
|  | IgG IE_SEV_ | -0.07 | (-0.12;-0.01) | **0.015** |  | -0.05 | (-0.11; 0.00) | 0.068 |
|  | IgG IE_ch1_ | 0.03 | (-0.04; 0.09) | 0.424 |  | 0.04 | (-0.03; 0.11) | 0.253 |
|  | IgG IE_ch2_ | -0.03 | (-0.11; 0.05) | 0.414 |  | -0.03 | (-0.11; 0.05) | 0.425 |
|  | IgM MSP | 0.03 | (-0.09; 0.15) | 0.610 |  | 0.03 | (-0.09; 0.15) | 0.610 |
|  | IgM EBA175 | -0.08 | (-0.17; 0.02) | 0.111 |  | -0.06 | (-0.15; 0.03) | 0.186 |
|  | IgM AMA | -0.01 | (-0.11; 0.09) | 0.861 |  | 0.02 | (-0.08; 0.12) | 0.749 |
|  | IgM DBLα | -0.01 | (-0.15; 0.14) | 0.939 |  | -0.04 | (-0.18; 0.11) | 0.606 |
|  | IgM lysate | -0.03 | (-0.06;-0.01) | **0.008** |  | -0.03 | (-0.05;-0.00) | **0.021** |
| **Maternal haemoglobin** | | |  |  |  |  |  |  |
|  | IgG MSP | -1.57 | (-5.21; 2.08) | 0.401 |  | -1.16 | (-4.91; 2.59) | 0.544 |
|  | IgG EBA175 | 0.61 | (-4.26; 5.49) | 0.806 |  | -0.14 | (-5.07; 4.79) | 0.956 |
|  | IgG AMA | 0.12 | (-5.47; 5.72) | 0.965 |  | -0.04 | (-5.77; 5.68) | 0.988 |
|  | IgG DBLα | 1.15 | (-6.11; 8.42) | 0.756 |  | 0.39 | (-7.22; 8.00) | 0.920 |
|  | IgG lysate | -0.22 | (-3.22; 2.77) | 0.885 |  | -0.41 | (-3.49; 2.67) | 0.794 |
|  | IgG CS2 | 0.66 | (-3.75; 5.07) | 0.771 |  | 0.32 | (-4.28; 4.91) | 0.893 |
|  | IgG IE_plac1_ | -0.68 | (-5.93; 4.58) | 0.801 |  | -0.37 | (-5.99; 5.25) | 0.897 |
|  | IgG IE_plac2_ | 0.00 | (-5.20; 5.20) | 0.999 |  | 0.28 | (-4.98; 5.53) | 0.918 |
|  | IgG IE_SEV_ | -0.67 | (-4.07; 2.74) | 0.701 |  | -0.85 | (-4.45; 2.74) | 0.643 |
|  | IgG IE_ch1_ | 2.30 | (-1.78; 6.38) | 0.271 |  | 2.58 | (-1.74; 6.89) | 0.244 |
|  | IgG IE_ch2_ | -4.38 | (-9.15; 0.38) | 0.073 |  | -4.05 | (-9.00; 0.90) | 0.111 |
|  | IgM MSP | -0.24 | (-7.97; 7.49) | 0.952 |  | -0.98 | (-8.80; 6.85) | 0.807 |
|  | IgM EBA175 | -4.01 | (-9.96; 1.93) | 0.188 |  | -4.25 | (-10.24; 1.74) | 0.167 |
|  | IgM AMA | -4.07 | (-10.42; 2.29) | 0.211 |  | -3.72 | (-10.22; 2.78) | 0.264 |
|  | IgM DBLα | 5.46 | (-3.94;14.86) | 0.257 |  | 7.11 | (-2.61;16.84) | 0.154 |
|  | IgM lysate | 0.45 | (-1.14; 2.05) | 0.577 |  | 0.37 | (-1.27; 2.02) | 0.658 |
| **Malaria incidence in children** | | |  |  |  |  |  |  |
|  | IgG MSP | 1.35 | (0.90; 2.02) | 0.143 |  | 1.34 | (0.88; 2.05) | 0.176 |
|  | IgG EBA175 | 1.50 | (0.88; 2.54) | 0.133 |  | 1.55 | (0.90; 2.70) | 0.117 |
|  | IgG AMA | 1.88 | (0.69; 5.17) | 0.219 |  | 1.78 | (0.66; 4.85) | 0.256 |
|  | IgG DBLα | 0.83 | (0.37; 1.87) | 0.652 |  | 0.85 | (0.34; 2.08) | 0.716 |
|  | IgG lysate | 0.97 | (0.64; 1.47) | 0.890 |  | 0.91 | (0.59; 1.40) | 0.665 |
|  | IgG CS2 | 1.10 | (0.69; 1.76) | 0.684 |  | 0.99 | (0.61; 1.61) | 0.981 |
|  | IgG IE_plac1_ | 1.15 | (0.72; 1.83) | 0.572 |  | 1.27 | (0.76; 2.11) | 0.360 |
|  | IgG IE_plac2_ | 0.98 | (0.60; 1.61) | 0.944 |  | 1.08 | (0.66; 1.79) | 0.750 |
|  | IgG IE_SEV_ | 1.23 | (0.85; 1.79) | 0.275 |  | 1.18 | (0.80; 1.75) | 0.394 |
|  | IgG IE_ch1_ | 1.00 | (0.64; 1.57) | 0.988 |  | 1.09 | (0.68; 1.74) | 0.713 |
|  | IgG IE_ch2_ | 1.07 | (0.57; 1.99) | 0.843 |  | 0.99 | (0.52; 1.90) | 0.976 |
|  | IgM MSP | 1.49 | (0.72; 3.08) | 0.282 |  | 1.6 | (0.75; 3.42) | 0.224 |
|  | IgM EBA175 | 0.59 | (0.32; 1.09) | 0.090 |  | 0.56 | (0.29; 1.08) | 0.084 |
|  | IgM AMA | 1.08 | (0.54; 2.18) | 0.824 |  | 0.98 | (0.46; 2.08) | 0.958 |
|  | IgM DBLα | 0.83 | (0.33; 2.06) | 0.680 |  | 0.84 | (0.33; 2.17) | 0.722 |
|  | IgM lysate | 0.88 | (0.72; 1.09) | 0.241 |  | 0.86 | (0.69; 1.06) | 0.156 |
|  | | | | | | | | |
